# Amputation of Tongue

**Published:** 1881-04

**Authors:** 


### CASE DEPARTMENT.

### COLUMBIA VETERINARY COLLEGE HOSPITAL.

### REPORTS OF CASES UNDER CARE OF FRANK WALTON, D. V. S., HOUSE SURGEON.

I.—AMPUTATION OF TONGUE.—A horse was brought to the hospital January 12th, 1881, for an extensive laceration of the tip of tongue and an almost complete division at the base. Owing to the exte ntof the anterior wound, it was thought best to excise the anterior two inches of the tongue, and to stitch the posterior laceration together. Five sutures were applied to the posterior wound, which closely approximated its lip. A cord was drawn tightly around the tongue, just posterior to the portion which was to be removed, and the tongue divided with one sweep of the knife. The cord was allowed to remain around the tongue for two hours; no secondary hemorrhage of any extent followed, although there was a little oozing of blood after removing the cord. The animal was nourished with semi-fluid food, and the mouth washed with deodorizing lotions. Both wounds healed rapidly, and the animal was discharged in thirteen days after admission to hospital, cured.

